# Using an Event-History with Risk-Free Model to Study the Genetics of Alcoholism

**DOI:** 10.1038/s41598-017-01791-4

**Published:** 2017-05-16

**Authors:** Hsin-Chou Yang, I-Chen Chen, Yuh-Chyuan Tsay, Zheng-Rong Li, Chun-houh Chen, Hai-Gwo Hwu, Chen-Hsin Chen

**Affiliations:** 1 Institute of Statistical Science, Academia Sinica, Taipei, 11529 Taiwan; 2 Department of Biostatistics, University of Kentucky, Lexington, KY 40506 USA; 3 Graduate Institute of Epidemiology and Preventive Medicine, National Taiwan University, Taipei, Taiwan; 4 Department of Psychiatry, National Taiwan University Hospital and College of Medicine, National Taiwan University, Taipei, Taiwan

## Abstract

Case–control genetic association studies typically ignore possible later disease onset in currently healthy subjects and assume that subjects with diseases equally contribute to the likelihood for inference, regardless of their onset age. Therefore, we used an event-history with risk-free model to simultaneously characterize alcoholism susceptibility and onset age in 65 independent non-Hispanic Caucasian males in the Collaborative Study on the Genetics of Alcoholism. Following data quality control, we analysed 22 single nucleotide polymorphisms (SNPs) on 12 candidate genes. The single-SNP analysis showed that the dominant minor allele of rs2134655 on *DRD3* increases alcoholism susceptibility; the dominant minor allele of rs1439047 on *NTRK2* delays the alcoholism onset age, but the additive minor allele of rs172677 on *GRIN2B* and the dominant minor allele of rs63319 on *ALDH1A1* advance the alcoholism onset age; and the dominant minor allele of rs1079597 on *DRD2* shortens the onset age range. Similarly, multiple-SNPs analysis revealed joint effects of rs2134655, rs172677 and rs1079597, with an adjustment for habitual smoking. This study provides a more comprehensive understanding of the genetics of alcoholism than previous case–control studies.

## Introduction

The identification of disease susceptibility genes is a primary step for genetic dissection of complex disorders^1^. Many statistical disease gene mapping methods (i.e., positional cloning) have been developed according to different phenotypes of interest (e.g., qualitative and quantitative traits), modes of inheritance (e.g., monogenic, oligogenic, and polygenic diseases), study designs (e.g., family- and population-based studies), and analysis strategy (e.g., linkage and association analyses)^2, 3^. One of the most common methods for a statistical gene mapping of complex disorders is a population-based case–control association study, which ensures convenient data collection and promising test power^4^.

Contingency table and logistic regression analyses^5^ have been widely applied to examine the genetic association, namely linkage disequilibrium (LD), of a dichotomous disease status with genetic markers in case–control genetic studies^6–8^. Using these methods to analyse a dichotomous disease event alone ignores the probability of later disease onsets in currently healthy subjects and incorrectly considers that all subjects with diseases equally contribute to the likelihood for inference, irrespective of differences in their onset age.

In addition to the disease status, we often acquire additional phenotype information of subjects, such as the age of onset, which provides the time to determine disease development in subjects. Event history analysis is commonly used to model the disease onset process^9, 10^. To date, the proportional hazards (PH) regression model^11^ has been the most widely used method. However, these methods implicitly assume that all subjects will eventually develop diseases. In fact, this may be erroneous because some subjects neither possess disease susceptibility genes nor have been exposed to harmful environments. Ignorance of the potential nonsusceptibility to the disease being studied may yield false conclusions. In this study, we used a novel event-history with risk-free model^12^ considering disease nonsusceptibility to determine the time to disease development. This method provides a useful alternative to traditional survival and event history analyses that do not consider nonsusceptibility^13^. Incorporation of nonsusceptibility into the event history model is to accurately define the denominator with potential subjects at risk in calculating the conditional disease probability at each time point.

The event-history with risk-free model is used to study the genetics of alcoholism. Alcoholism, a complex disorder, has a multifactorial and polygenic mode of inheritance^14^. In Caucasian populations, the 12-month and lifetime prevalences of alcohol dependence were 3.8% and 13.8%, respectively^15^. A twin study revealed that the genetic heritability of alcoholism was between 40% and 60% in Caucasian populations^16^. Some disease susceptibility genes for alcoholism have been identified^17, 18^; however, almost all genetic studies have only considered the disease status instead of the event history of alcoholism as the endpoint. Essentially, the probability of susceptibility estimated in the event-history with risk-free model can be interpreted as the alcoholism lifetime prevalence. Provided that the alcoholism lifetime prevalence is neither too low nor too high, the event-history with risk-free model has its strength in studying genetics of alcoholism.

To identify specific susceptibility genes for alcoholism, the Collaborative Study on the Genetics of Alcoholism (COGA)^19^ collected data from more than 300 extended families, in which many members were affected by alcoholism. We analysed the susceptibility and age of onset of alcoholism by using the COGA data; however, the event-history with risk-free model^12^ currently only considers independent study subjects. Considering the effects of ethnic heterogeneity and sex difference on alcoholism, we hence focused on non-Hispanic Caucasian male founders in the COGA with the candidate gene approach by analysing single nucleotide polymorphisms (SNPs). In total, we enrolled 65 subjects in this study after excluding one subject with an age of onset of 91 years.

The 65 study subjects included 23 alcoholism cases and 42 unaffected subjects. For our event-history data analysis, phenotypic, genetic and environmental data of all subjects were available, except for a few missing SNP data. First, the age of onset for alcoholism cases and age at interview for unaffected subjects were considered a quantitative trait. The age at interview of unaffected subjects was considered a surrogate for the disease onset censoring time in our analysis. Second, the habitual smoking status was considered an environmental covariate. A habitual smoker was defined as a person who smoked ≥1 pack(s) a day for ≥6 months. In total, we included 46 habitual and 19 nonhabitual smokers in the proportion 19/23 and 27/42 of habitual smokers in the alcoholism case and unaffected groups, respectively. Finally, SNP markers were used for a genetic evaluation of disease susceptibility and the age of onset.

**Figure 1:**
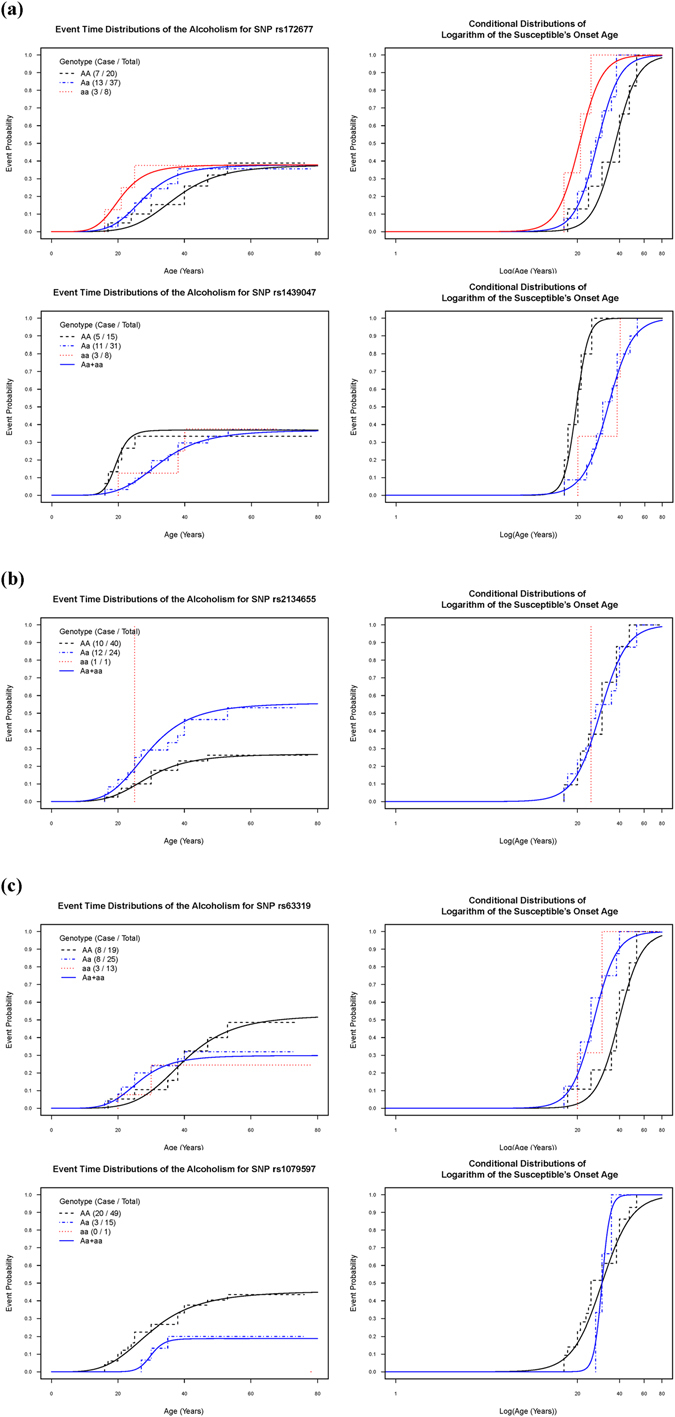
Kaplan–Meier (step function) and mixture regression (solid smooth curve) estimators of overall and conditional event curves for alcoholism onset age stratified by genotypes of a single SNP. (**a**) SNPs with an equal probability of susceptibility only: rs172677 and rs1439047, (**b**) SNPs with unequal probabilities of susceptibility only: rs2134655, (**c**) SNPs with unequal susceptibilities and onset age: rs63319 and rs1079597. Note that all the Kaplan–Meier curves are stratified by genotypes *AA*, *Aa* and *aa* while estimated mixture regression curves are stratified by genotypes *AA* and *Aa* + *aa* except the SNP rs172677.

Corresponding to some SNPs of the COGA, all Kaplan–Meier event curves^20^ estimated for the onset age (Fig. 1, left panel) showed flat tails not increasing to 1.0 at a later age. We termed the fraction on the flat tail as the probability of susceptibility. Right-censored subjects, without diseases during the follow-up period, would either be affected in later years or would remain nonsusceptible to diseases throughout their lifetime. No genetic study of alcoholism has utilized a regression model that simultaneously considers nonsusceptibility and age of onset. In this study, we used a logistic-accelerated failure time (AFT) location-scale mixture regression model^12^ to explore respective crucial genetic and environmental effects for the probability of developing alcoholism and age of onset in susceptible cases.

## Results

### Candidate genes

We collected information on alcoholism-associated genes from two established public-domain disease databases, Online Mendelian Inheritance in Man (OMIM)^21^ and Genetic Association Database (GAD)^22^. In total, we obtained 15 genes from OMIM (see Supplementary List S1) and 65 genes from GAD (see Supplementary List S2). The intersection and union of gene sets from OMIM and GAD contained 10 and 70 candidate genes, respectively.

We used the dbSNP of the National Center for Biotechnology Information^23^ to map the SNPs to a union list of 70 genes. As shown in Supplementary Tables S1 and S2, 31 SNPs from the COGA data set can be mapped to the following 14 genes from both databases: *ALDH1A1* (rs63319 and rs348457), *CHRM2* (rs1378647 and rs1111418), *DRD2* (rs1079598, rs1079597, and rs1079596), *DRD3* (rs2134655), *FYN* (rs1409836 and rs910683), *GABRA1* (rs966137 and rs1157122), *GABRB1* (rs728293 and rs956412), *GABRB3* (rs1365225), *GABRG2* (rs411409, rs387661, and rs2422106), *GRIN2B* (rs172677 and rs1421109), *HTR2A* (rs985934, rs985933, and rs927544), *NTRK2* (rs1439047 and rs1838158), *RXRG* (rs2134095 and rs157864), and *TPH2* (rs1386493, rs1386492, rs1386485, and rs1386483). Except the SNP rs2134095 located in a coding-synonymous region, all other SNPs were located in intronic regions.

### SNP selection

Based on the 65 samples, we calculated allele frequencies, genotype frequencies, and the genotyping call rate (GCR). We excluded SNPs with a low minor allele frequency (MAF < 0.05) and GCR (<85%). The results revealed that rs1409836 and rs910683 were nonpolymorphic with an MAF of 0 and 0.008, respectively, and were excluded from this study. Therefore, the gene *FYN* was excluded from this study. In addition, rs1421109 on *GRIN2B* was excluded because of its low MAF (0.031) and GCR (73.8%; Supplementary Table S1).

**Figure 2:**
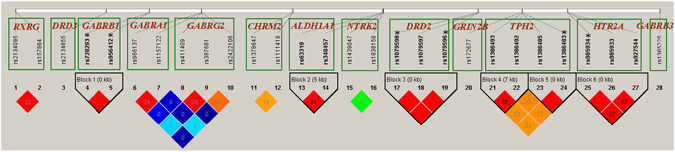
LD plot of 28 SNPs on 13 candidate genes. In the LD heat map, 13 genes are arranged according to their chromosomes. SNPs (RS numbers) within the same gene are arranged by physical positions and framed by a green rectangle. Pairwise LD of SNPs within the same gene was measured using D′ and r^2^ ^50^. The colored rhombus reflects the D′ magnitude (higher LD, red) and the numbered rhombus indicates the r^2^ value. SNPs with a strong LD are framed in a black inverse diamond block, which is defined according to the confidence interval method^51^. Six SNPs with a star sign concatenated with the RS number are removed from the subsequent analysis because of a complete LD and/or HWE violation.

LD and LD blocks of the remaining 28 SNPs on 13 candidate genes were plotted using Haploview^24^ (Fig. 2). Several SNPs were in complete LD with other SNPs in the same LD block; therefore, we eliminated the SNPs having a lower GCR, namely rs728293, rs1079598, rs1079596, rs1386483, and rs985934. We performed the Hardy–Weinberg equilibrium (HWE) test in the unaffected group by using Haploview. The SNPs rs728293 and rs956412 on *GABRB1* violated the HWE with p values of 0.029 and 0.032, respectively. The other SNPs fitted the HWE. The elimination of rs728293 and rs956412 resulted in the exclusion of *GABRB1* in this study. In summary, we eliminated nine SNPs (Supplementary Table S1) and focused on 22 SNPs on 12 candidate genes (Supplementary Table S2) in the subsequent analysis.

### Analysis strategy of susceptibility and age at onset

Considering genotypes of each of the 22 studied SNPs, we examined whether they have (a) an equal probability of alcoholism susceptibility but different conditional age-of-onset distributions for subjects susceptible to alcoholism, (b) unequal probabilities of alcoholism susceptibility but the same conditional alcoholism age-of-onset distribution, (c) unequal probabilities of alcoholism susceptibility and different conditional alcoholism age-of-onset distributions, and (d) no overall difference. SNPs are classified into four exhaustive categories according to the mutually exclusive significances of association tests in the logistic regression submodel of Equation (2) and the AFT location-scale regression submodel of Equation (3). By using the same strategy, we also studied the effect of habitual smoking effect on the development of alcoholism.

Fitting the mixture regression model to each of the 22 studied SNPs (Supplementary Table S2), we identified that rs172677 and rs1439047 belonged to the aforementioned category (a), rs2134655 belonged to category (b), and rs63319 and rs1079597 belonged to category (c) showed some significant effects on alcoholism. By contrast, the genotypes of the remaining 17 SNPs belonging to category (d) showed nonsignificant effects on alcoholism. The regression results and event curves of genotype analysis corresponding to all five significant SNPs are presented in Table 1(A) and Fig. 1 with the following details. For testing the significance of all the regression parameters in the fitted mixture regression model versus the model with only the intercept term in all the regression parts, chi-squared statistics, degrees of freedom and corresponding p values of the likelihood ratio test are shown in the last three columns of Table 1(A)

**Table 1:** (A) Single-SNP analysis by using the logistic-AFT mixture regression model. (B) Corresponding bootstrap validation results based on 400 bootstrap samples of size 65.

SNP [Gene] (# Subjects)^a^	Covariates (Genotypes)	Logistic Regression Submodel			AFT Submodel (Log-logistic Event Time Distribution)						LRT^b^ for Mixture Model		
					Location Regression Part			Scale Regression Part					
		OR	95% CI	p-value	Estimate	95% CI	p-value	Estimate	95% CI	p-value	${{\rm{\chi }}}^{2}$	d.f.	p-value
**(A) Original Fitting**													
rs172677 [*GRIN2B*] (65)	Intercept	1		Referent (*Aa*)	3.33	3.15, 3.51	Referent	−1.69	−2.07, −1.31	Referent	6.22	2	0.045
	*aa*	1			−0.31	−0.69, 0.07	0.107	0					
	*AA*	1			0.28	−0.04, 0.61	0.087	0					
rs172677 [*GRIN2B*] (65)	Intercept	1		Referent (*AA*)	3.62	3.38, 3.86	Referent	−1.69	−2.07, −1.32	Referent	6.22	1	0.013
	*a* (*Aa* = 1, *aa* = 2)	1			−0.29	−0.51, −0.08	0.006	0					
rs1439047 [*NTRK2*] (54)	Intercept	1		Referent (*AA*)	2.97	2.82, 3.12	Referent	−2.34	−3.05, −1.63	Referent	10.73	2	0.005
	*Aa* + *aa*	1			0.52	0.27, 0.76	<0.001	0.74	−0.13, 1.61	0.093			
rs2134655 [*DRD3*] (65)	Intercept	1		Referent (*AA*)	3.37	3.20, 3.54	Referent	−1.52	−1.91, −1.13	Referent	4.87	1	0.027
	*Aa* + *aa*	3.44	1.12, 10.59	0.031	0			0					
rs63319 [*ALDH1A1*] (57)	Intercept	1		Referent (*AA*)	3.68	3.43, 3.93	Referent	−1.67	−2.11, −1.24	Referent	6.81	2	0.033
	*Aa* + *aa*	0.38	0.10, 1.49	0.166	−0.41	−0.73, −0.10	0.010	0					
rs1079597 [*DRD2*] (65)	Intercept	1		Referent (*AA*)	3.40	3.28, 3.52	Referent	−1.40	−1.82, −0.98	Referent	7.14	2	0.028
	*Aa* + *aa*	0.28	0.07, 1.12	0.073	0			−1.32	−2.34, −0.30	0.011			
**(B) Bootstrap Validation**													
rs172677 [*GRIN2B*] (65)	Intercept	1			3.61	3.30, 3.85		−1.77	−2.15, −1.44				
	*a* (*Aa* = 1, *aa* = 2)	1			−0.29	−0.50, −0.02		0					
rs1439047 [*NTRK2*] (54)	Intercept	1			3.02	2.82, 3.50		−2.53	−3.93, −0.95				
	*Aa* + *aa*	1			0.47	0.05, 0.78		0.86	−0.56, 2.29				
rs2134655 [*DRD3*] (65)	Intercept	1			3.38	3.20, 3.57		−1.56	−1.91, −1.24				
	*Aa* + *aa*	4.49	1.24, 12.56^c^		0			0					
rs63319 [*ALDH1A1*] (57)	Intercept	1			3.65	3.39, 3.85		−1.77	−2.20, −1.39				
	*Aa* + *aa*	0.51	0.07, 1.63		−0.37	−0.65, −0.06		0					
rs1079597 [*DRD2*] (65)	Intercept	1			3.40	3.25, 3.53		−1.44	−1.86, −1.15				
	*Aa* + *aa*	0.36	0.09, 0.92		0			−1.39	−2.23, −0.03				

^a^The sample size for each single-gene analysis. ^b^Chi-squared statistic, degrees of freedom and p-value for the likelihood ratio test. ^c^bootstrap percentile confidence intervals. Abbreviations: OR, odds ratio; CI, confidence interval; LRT, likelihood ratio test; d.f., degrees of freedom.

.

### Single-SNP analysis

#### With an equal probability of susceptibility but different conditional age-of-onset distributions

The logistic regression submodel estimates that three genotypes of rs172677 have the same probability of alcoholism susceptibility (0.378). By contrast, the negative and positive parameter estimates of −0.31 and 0.28, respectively, in the location regression part of rs172677 with *Aa* as the referent genotype indicates that susceptible subjects with genotypes *aa* and *AA* tend to have an earlier and later age of onset of alcoholism, respectively (p = 0.107 vs 0.087). A similar magnitude of these two estimated location parameters suggests an additive effect of allele *a* on the age of onset of alcoholism. By fitting the mixture regression with the additive model of the location regression part, we confirmed the additive effect of allele *a* with significance (p = 0.006). This analysis affirms that the three genotypes of rs172677 have an equal probability of alcoholism susceptibility; however, susceptible subjects with the harmful additive allele *a* would have an earlier age of onset of alcoholism. By using the additive model, the median onset age of susceptible subjects with genotypes *aa*, *Aa*, and *AA* were estimated to be 20.7, 27.8, and 37.3 years, respectively.

Three genotypes of rs1439047 were merged into two subgroups, *Aa* + *aa* and *AA*, to represent the dominant model for allele *a*. These two subgroups showed the same estimated probability of alcoholism susceptibility (0.369). Compared with the referent genotype *AA*, the positive estimate 0.52 of the location difference for genotype *Aa* + *aa* in the AFT log-logistic model indicated that susceptible subjects with allele *a* of rs1439047 would have a later age of onset of alcoholism (p < 0.001), whereas the corresponding estimated scale difference of 0.74 indicated a larger age range (p = 0.093). These results showed that the dominant allele *a* had a preventive effect on delaying the age of onset of alcoholism by 13.2 (=32.7–19.5) years of the median onset age in susceptible subjects despite its susceptibility to alcoholism being the same as that in genotype *AA*.

#### With unequal probabilities of susceptibility but the same conditional age-of-onset distribution

We considered the dominant model for allele *a* of rs2134655 on the basis of the referent genotype *AA* in the logistic regression submodel and obtained the corresponding estimated OR = 3.44 (p = 0.031) as the only significant parameter in mixture regression, with an identical estimated conditional age-of-onset distribution among all genotypes of rs2134655. An increase in the probability of susceptibility because of the harmful allele *a* was estimated to be 0.29.

#### With unequal probabilities of susceptibility and different conditional age-of-onset distributions

Analysis on the basis of two subgroups of the merged genotypes in both rs63319 and rs1079597 resulted in both unequal probabilities of susceptibility and different conditional age-of-onset distributions of alcoholism between the subgroups. First, the dominant allele *a* of rs63319 showed a lower alcoholism susceptibility, with an estimated OR of 0.38 (p = 0.166), corresponding to a decrease of 0.23 in the probability of susceptibility. However, allele *a* showed a significantly earlier alcoholism onset age, with an estimated location difference of −0.41 (p = 0.010), rendering an onset age 13.4 (=39.6–26.2) years earlier in the estimated median onset age of susceptible subjects. Second, the estimated OR = 0.28 (p = 0.073) with respect to the dominant allele *a* of rs1079597 suggests a preventive effect on reducing the probability of susceptibility by 0.33, without a difference in location parameters; however, the estimated scale difference of −1.32 (p = 0.011) signifies a shorter range of the alcoholism onset age associated with allele *a*.

Overall speaking, Fig. 1, demonstrate adequate fitting of the logistic-AFT log-logistic location-scale mixture model to the identified SNPs.

### Multiple–SNPs analysis

In rs1439047 and rs63319, 11 and 8 observations were missing, respectively, which would result in smaller sample sizes in some genotypic combinations. Therefore, to evaluate multiple gene effects on alcoholism in the 65 subjects, we considered only the three SNPs, rs172677, rs2134655, and rs1079597, among the five identified SNPs. Because of the limited sample size, we fit the subset regression models by including only the main effects of the three selected SNPs. The regression result of the best-fitted model with multiple genes is presented in Table 2. Similar to the patterns in the single-SNP analysis, the results demonstrated that the dominant allele *a* of rs2134655 had a higher probability of alcoholism susceptibility than did genotype *AA* with an estimated OR of 3.14 (p = 0.059). The additive allele *a* of rs172677 showed an earlier alcoholism onset age than did genotype *AA* (p = 0.020). The dominant allele *a* of rs1079597 showed a shorter range of the alcoholism onset age than did genotype *AA*

**Table 2:** Multiple-SNPs analysis by using the logistic-AFT mixture regression model with chi-squared statistic 15.48 (p = 0.004) of the likelihood ratio test in the mixture model.

SNP [Gene]	Covariates (Genotypes)	Logistic Regression Submodel			AFT Submodel (Log-logistic Event Time Distribution)					
					Location Regression Part			Scale Regression Part		
		OR	95% CI	p-value	Estimate	95% CI	p-value	Estimate	95% CI	p-value
rs2134655 [*DRD3*]	Intercept	1		Referent						
	*Aa* + *aa*	3.14	0.96, 10.32	0.059						
rs172677 [*GRIN2B*]	Intercept				3.66	3.40, 3.92	Referent			
	*a* (*Aa* = 1, *aa* = 2)				−0.29	−0.53, −0.04	0.020			
rs1079597 [*DRD2*]	Intercept							−1.55	−1.99, −1.11	Referent
	*Aa* + *aa*	0.36	0.08, 1.52	0.164				−1.13	−2.24, −0.03	0.044

Abbreviations: OR, odds ratio; CI, confidence interval.

(p = 0.044). The likelihood ratio test with p = 0.004 indicates an adequate fitting with multiple SNPs.

### Environmental covariate analysis

The habitual smoking status may be a potential environmental covariate in studying modified genetic effects on alcoholism. We first considered habitual smoking as a single factor in the mixture regression model; the regression results and event curves are shown in Supplementary Table S3 and Supplementary Figure S1. The Kaplan–Meier event curves^20^ display that habitual smokers had a higher probability of alcoholism susceptibility than did nonhabitual smokers. However, the mixture model identified habitual smoking as nonsignificant in the logistic regression submodel and only a significant location parameter of −0.47 (p = 0.046), indicating an earlier onset age caused by habitual smoking.

**Figure 3:**
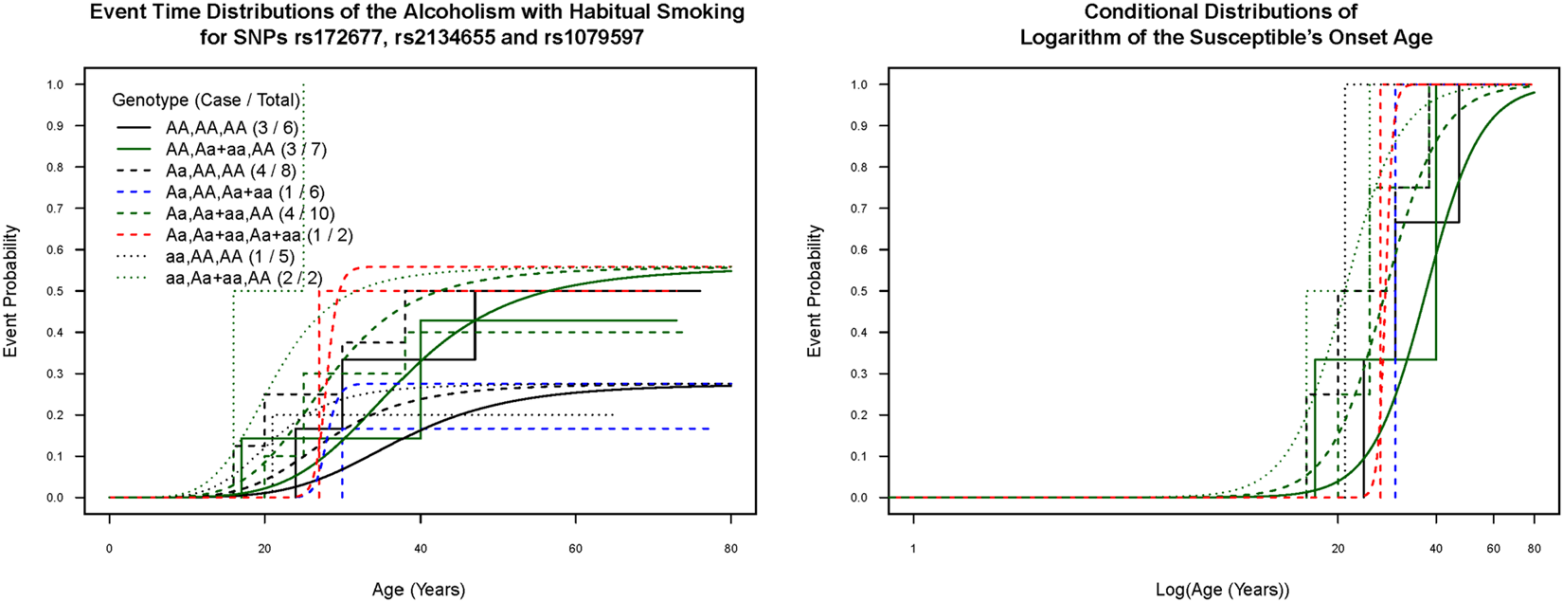
Kaplan–Meier (step function) and multiple mixture regression (smooth curve) estimators of overall and conditional event curves in all habitual smokers for the alcoholism onset age by genotypes of three SNPs and habitual smoking.

We conducted further adjustment for the habitual smoking status with the three selected SNPs rs172677, rs2134655, and rs1079597 from the best-fitted multiple-SNPs model and fit the subset regression models with the main effects of habitual smoking and the three SNPs. The regression result and estimated event curves of the best-fitted model with multiple SNPs and the environmental factor are presented in Table 3 and Fig. 3. Similar to the previous multiple-SNPs analysis, the inclusion of the habitual smoking status in the location regression part retains almost the same patterns as the three SNPs. The dominant allele *a* of rs2134655 showed a significantly higher probability of alcoholism susceptibility than did genotype *AA*, with an estimated OR of 3.33 (p = 0.037); both additive allele *a* of rs172677 and habitual smoking showed effects on an earlier alcoholism onset age (p = 0.002 vs <0.001). The dominant allele *a* of rs1079597 showed a shorter range of the alcoholism onset age than did genotype *AA* (p < 0.001). Among the gene–environment strata, susceptible habitual smokers with genotype *aa* of rs172677 demonstrated the earliest alcoholism onset age, whereas susceptible nonhabitual smokers with genotype *AA* of SNP rs172677 showed the latest onset age. The likelihood ratio test with p < 0.001 demonstrates a much better fitting of the gene–environment model than of the three-SNPs model. Because of the high proportion of habitual smokers and only four alcoholism cases of 19 nonhabitual smokers with sparse events in SNP strata, we plotted Fig. 3 corresponding to the three SNPs in the 46 habitual smokers and omitted nonhabitual smokers from the figure because they contributed at most one alcoholism event in each stratum. In Fig. 3, the combined effects of the three SNPs and habitual smoking provide fitted overall and conditional alcoholism age-of-onset distributions much close to the corresponding Kaplan–Meier event curves^20^

**Table 3:** Analysis of multiple SNPs and habitual smoking by using the logistic-AFT mixture regression model with chi-squared statistic 21.30 (p < 0.001) of the likelihood ratio test in the mixture model.

Factors SNP [Gene]	Covariates	Logistic Regression Submodel			AFT Submodel (Log-logistic Event Time Distribution)					
					Location Regression Part			Scale Regression Part		
		OR	95% CI	p-value	Estimate	95% CI	p-value	Estimate	95% CI	p-value
rs2134655 [*DRD3*]	Intercept	1		Referent (*AA*)						
	*Aa* + *aa*	3.33	1.08, 10.29	0.037						
rs172677 [*GRIN2B*] and HS	Intercept				3.85	3.67, 4.03	Referent			
	*a* (*Aa* = 1, *aa* = 2)				−0.29	−0.47, −0.11	0.002			
	HS				−0.23	−0.33, −0.13	<0.001			
rs1079597 [*DRD2*]	Intercept							−1.64	−2.05, −1.22	Referent
	*Aa* + *aa*							−1.97	−3.03, −0.92	<0.001

Abbreviations: HS, Habitual smoking (Yes = 1, No = 0); OR, odds ratio; CI, confidence interval.

in all strata of habitual smokers.

**Figure 4:**
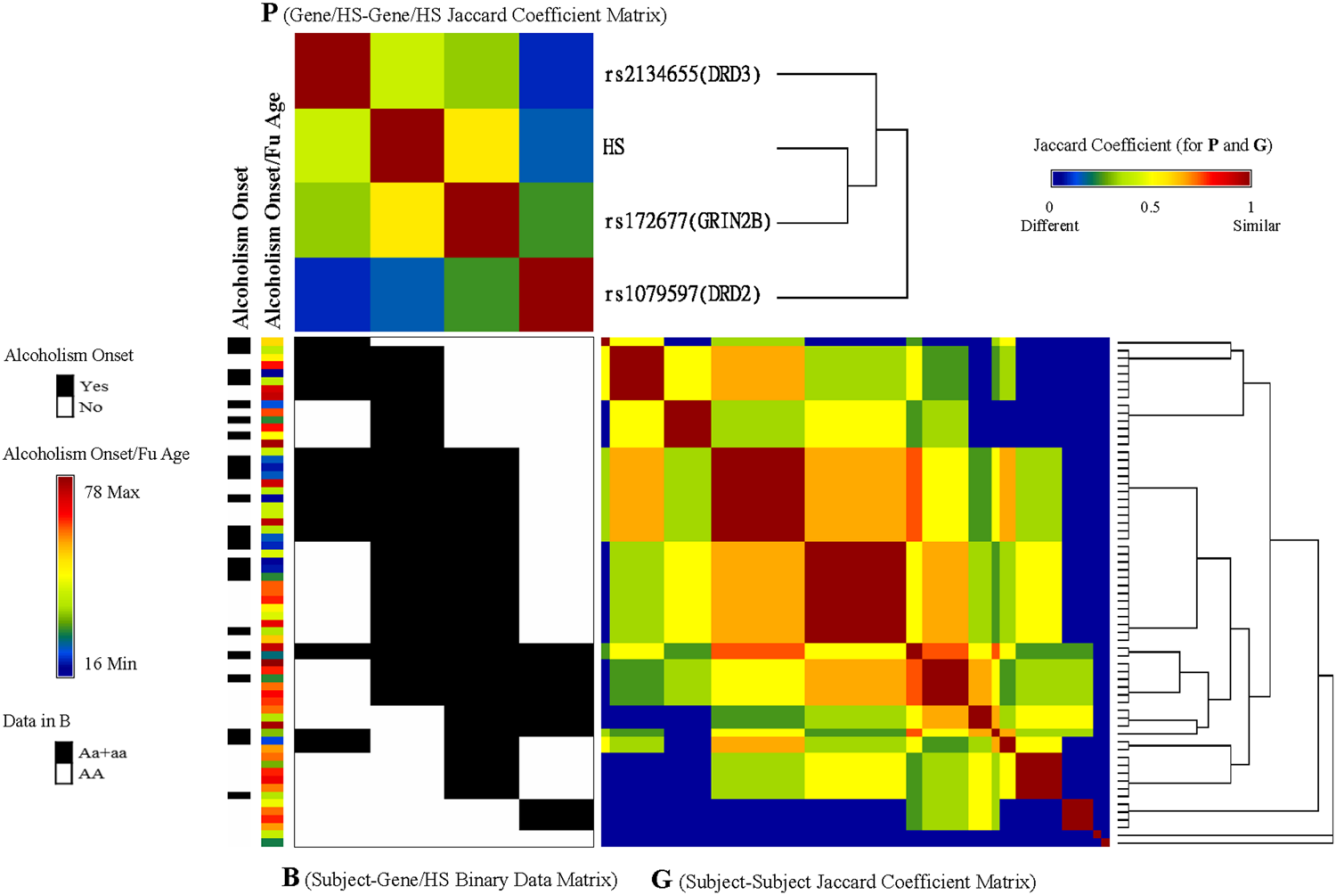
GAP clustering of three multiple SNPs (genes) and habitual smoking of 65 subjects.

To examine the associations among the gene–environment strata fitted in Table 3, we plotted Fig. 4 to display the clustering of the three selected SNPs (genes) and habitual smoking of the 65 subjects by using Generalized association plots (GAP)^25, 26^. Considering the minor-allele carriers as risky revealed that *GRIN2B* (rs172677) highly overlaps with the habitual smoking status. In addition, smokers who carried the risky allele on *GRIN2B* tended to have an early alcoholism onset than those on *DRD3* (rs2134655). Moreover, the clustering of the 65 subjects into several subgroups based on the three SNPs and habitual smoking can be observed from the matrix **G** of between-subject associations.

### Pathway analysis

**Figure 5:**
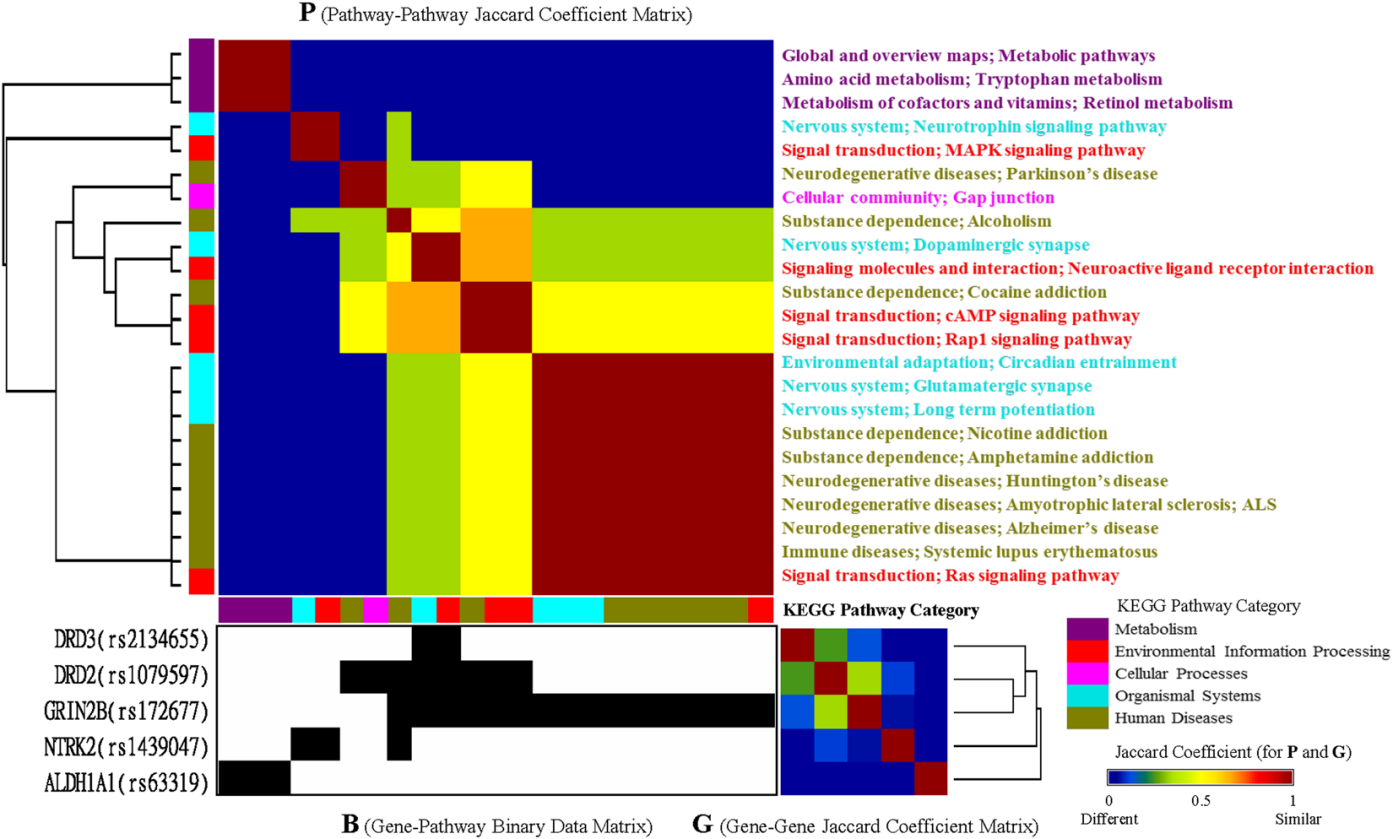
GAP clustering of the pathways of five identified significant genes from the KEGG for alcoholism.

Genes harboring the identified SNPs associated with alcoholism risks may interconnect in biological pathways. Using gene annotation from the Kyoto Encyclopedia for Genes and Genomics (KEGG)^27^, we analysed pathways related to five alcoholism genes, which contain the five identified SNPs (Table 1) significantly associated with risks and age at onset of alcoholism in the single-SNP analysis. GAP analysis^25, 26^ in Fig. 5 displays the clustering of five candidate genes and the relevant 23 pathways, with five categories of the KEGG^27^ coded in five colors.

The dendrogram of the five genes for the Jaccard coefficient matrix **G** (Fig. 5) on the basis of 23 KEGG pathways demonstrated that *DRD2* (rs1079597) and *GRIN2B* (rs172677) show the highest similarity with six common pathways belonging to three categories: human diseases (alcoholism and cocaine addiction) in dark yellow, organismal systems (dopaminergic synapse) in cyan, and environmental information processing [neuroactive ligand–receptor interaction and cyclic adenosine monophosphate (cAMP) and Rap1 signaling] in red. Moreover, clustering of *DRD2* (rs1079597) and *GRIN2B* (rs172677) was associated with *NTRK2* (rs1439047) and *DRD3* (rs2134655), respectively. The cluster of *DRD2* and *GRIN2B* share the alcoholism pathway with *NTRK2* (rs1439047) and have the same pathways of neuroactive ligand–receptor interaction and dopaminergic synapse as does *DRD3* (rs2134655). However, no association was observed between *ALDH1A1* (rs63319), which is only involved in the metabolism category, and the remaining four genes.

The dendrogram for the Jaccard coefficient matrix **P** among the 23 KEGG pathways on the basis of the five genes and the sorted data matrix **B** in Fig. 5 reveal that the pathway pair of dopaminergic synapse and neuroactive ligand–receptor interaction (sharing *DRD3*, *DRD2*, and *GRIN2B*) is strongly associated with the pathway group of cocaine addiction, cAMP signaling, and Rap1 signaling (sharing *DRD2* and *GRIN2B*). In addition to the aforementioned pathway group, the alcoholism pathway shares *DRD2* and *GRIN2B*. *GRIN2B* is shared by all 10 pathways (three organismal systems, six human diseases, and one environmental information processing pathway) in the largest pathway group. In summary, the six overlapping pathways among the four genes are correlated.

## Discussion

The logistic regression model is one of the most common statistical methods in case–control genetic association studies but tends to lose information if the age of onset is available. The logistic-AFT location-scale mixture regression model^12^ can simultaneously determine the probability of disease susceptibility and distribution of the age of onset for susceptible subjects. In principle, this flexible mixture model provides detailed interpretations and a more comprehensive understanding of the genetics of diseases than does logistic regression analysis in a case–control study.

To demonstrate the advantage of the mixture regression model over the logistic regression model as in conventional association studies, we manipulated univariate logistic regression analysis for each identified SNP as shown in left panel of Supplementary Table S4. The results revealed that rs172677, rs1439047, rs63319, and rs1079597, but not rs2134655, had an equal probability of alcoholism susceptibility among genotypes. The merged genotype *Aa* + *aa* of rs2134655 was associated with a risk of alcoholism compared with genotype *AA* (p = 0.027). For each SNP, the conventional logistic regression analysis yielded the same conclusions as did the mixture regression analysis; in brief, the regression coefficient estimates in both conventional logistic model and logistic susceptibility submodel of the logistic-AFT mixture model are very close to each other. However, the logistic-AFT mixture model provided different interpretations in correctly combining susceptible and nonsusceptible subpopulations, with detailed information on the alcoholism onset age of susceptible subjects by using a flexible time-to-event model with location and scale regression parts.

A recent simulation study of the cure survival mixture model^13^ evaluated the performance of logistic regression and traditional PH analyses^11^ by using a mixture model and studied the advantages of mixture survival analysis. Their results demonstrate that in the presence of a cure fraction, both logistic and Cox PH regression analyses render biased regression estimates, and the Cox PH regression may even incorrectly identify the age of onset effects that do not exist. However, the mixture analysis provides unbiased estimates in the absence of a background genetic risk of other SNPs. Moreover, the simulation scenarios in the paper^13^ based on the logistic-Weibull mixture model in the presence of a background genetic risk reveal that the mixture analysis may result in the underestimation of both logistic and onset age effects by 12–15%, relatively independent of the nonsusceptibility fraction, as shown in their Fig. 6(b) and (d)^13^. In our mixture analysis with multiple SNPs, we did not adjust p values as in multiple testing problems. In accordance with the underestimation findings by Stringer *et al*.^13^, we allowed a threshold p value of slightly higher than 0.05 for selecting SNPs in each regression part of the mixture model.

In most previous genetic studies of alcoholism, the age of onset or age at interview was solely used to determine early or late-onset alcoholism for a stratified analysis^28^ or treated as a confounder in the linkage analysis of alcoholism^29, 30^. Few studies have formally used the age of onset or age at interview as the phenotype of interest in a statistical model. Liu *et al*. reported that narrow-sense heritability of the age of onset of alcoholism is approximately 38%^31^. Li *et al*. performed a rank-based association test to analyse the age of onset of alcoholism^32^. To analyse the age of onset of alcoholism in the COGA data set, Tayo *et al*. used the Cox PH model^33^, Zhong and Zhang employed additive genetic gamma frailty PH models^34^, Zhao implemented an extended Cox model with mixed effects and familial relationships^35^, and Kapoor *et al*. incorporated a clustered sandwich estimator in the Cox PH model to account for familial correlations^36^. However, these survival and event history analyses did not consider the potential phenomenon of nonsusceptibility.

To demonstrate the inadequacy of Cox PH model^11^ for the analysis of alcoholism in the COGA data, we also manipulated univariate Cox PH analysis for each identified SNP as shown in right panel of Supplementary Table S4. The results revealed the same alcoholism age-of-onset distributions among the genotypes of rs172677, rs1439047, rs63319 and rs1079597, respectively, and the only significant effect on alcoholism identified by the Cox PH model due to the dominant allele *a* of rs2134655 versus genotype *AA* with a hazard ratio of 2.45 (p = 0.034). These results from the Cox PH model contradicted the Kaplan-Meier curves^20^ in Fig. 1 since the PH assumption implies that the event curves cannot be crossing. The Cox PH model is sensitive to detect the difference of onset ages between genotypes of rs2134655 on *DRD3* since the corresponding overall event curves are not crossing; however, the mixture model presents novel interpretations that genotypes of rs2134655 cause different probabilities of susceptibility to alcoholism, but with the same onset-age distribution for the susceptible cases. The crossing patterns emerging in overall or conditional genotype-specific event curves for the other four SNPs lead to non-PH models which may be well fitted by the location-scale cure mixture model^12^.

In this study, the event-history with risk-free model^12^ allows analysing nonsusceptible subjects. On the basis of this general model, we identified rs2134655 on *DRD3* with unequal probabilities of alcoholism susceptibility (p = 0.027). Importantly, we are the first to report SNPs associated with unequal age-of-onset distributions for susceptible cases to alcoholism [rs172677 on *GRIN2B* (p = 0.013), rs1439047 on *NTRK2* (p = 0.005), rs63319 on *ALDH1A1* (p = 0.033), and rs1079597 on *DRD2* (p = 0.028)].

On the basis of single gene analyses of alcoholism, we conducted pathway analysis that revealed that the alcoholism pathway is associated with *DRD2*, *GRIN2B*, and *NTRK2* and the cocaine addiction pathway is associated with *DRD2* and *GRIN2B*. *DRD2* is involved in behavior rewarding and its dysfunction leads to aberrant substance-seeking behavior^17^. Our identified TaqI B (rs1079597) polymorphism on *DRD2* had a higher allele frequency of B1 (i.e., minor allele *a* in this study) in a Caucasian severe alcoholic group than in nonalcoholic and less severe alcoholic groups^37^. *GRIN2B* is a glutamate-gated ion channel^38^ involved in memory and learning, and mutations in *GRIN2B* result in a severe neurodevelopmental phenotype with mental retardation^39^. Increasing evidence suggests that the N-methyl-D-aspartate type of glutamate receptor contributes to the development of alcoholism and withdrawal symptoms when alcohol intake is ceased^40^. Rs2134655 on *DRD3* identified to be associated with the alcoholism susceptibility is related to the signal transduction of cAMP and Rap1 pathways. *GRIN2B*, *DRD2*, and *DRD3* participate in the dopamine pathway and neuroactive ligand–receptor interaction pathway. In addition to *NTRK2*, these three genes seem to act cooperatively to induce substance-dependent behaviors, including those caused by alcoholism, cocaine addiction, and others. *ALDH1A1* belongs to the KEGG pathway of metabolism. *ALDH1A1* is associated with alcoholism as it confers protective effects against alcoholism caused by the deficiency of aldehyde dehydrogenase in aldehyde metabolism and results in an extremely high aldehyde level in the blood, thus causing aversive toxic and skin flushing responses in Caucasians^41^. Thus, the mechanism underlying *ALDH1A1*-related alcoholism is different from the gene cluster of *DRD2*, *DRD3*, *GRIN2B*, and *NTRK2*, which is directly involved in behavioural rewarding effects of alcohol consumption. Notably, the five alcoholism-associated genes reported in this study form different gene clusters in the GAP gene-pathway clustering analysis. The *DRD2*–*GRIN2B*–*NTRK2* and *GRIN2B*–*DRD2*–*DRD3* gene clusters function in synchrony to facilitate the development of alcoholism in subjects having the specific DNA polytheisms of these genes.

Most previous association studies of alcoholism relied on categorical data analysis by treating alcoholism as a dichotomous disease status. Some studies were claimed as age-of-onset studies, including an association study of TaqI B on *DRD2* with an early age of onset (≤12 years) of alcohol consumption in Mexican Americans^42^. The onset of alcoholism essentially spans many years; therefore, the event-history with risk-free model implemented in this study provides more realistic interpretations than does any categorical data analysis. In particular, modelling with the probabilities of susceptibility and conditional age-of-onset distributions provides separate interpretations of long- and short-term effects resulting from genetic and environmental factors. This study presents different roles played by the identified genes: *DRD3* was associated with the long-term lifetime risk of alcoholism; *GRIN2B* acting independently with the habitual smoker had additive effects on age of onset of alcoholism for susceptible cases; *DRD2* was related to the early alcoholism onset in view of a short age range. They necessitate further biomedical validations.

A small sample size is the limitation of this study. This study analysed only 65 subjects from the Genetic Analysis Workshop 14 (GAW14) data set that contained 1614 subjects from 14 large pedigrees. First, no statistical models have been developed for simultaneously studying disease susceptibility and age of onset under a family-based genetic association study, although some software (e.g., PENETRANCE in MENDEL)^43^ can handle age of onset for penetrance estimation and the subsequent association analyses, and some other software (e.g., SEGREG in S.A.G.E.)^44^ can jointly analyze the susceptibility and age of onset for segregation analyses. Hence, we only selected independent founders from the 14 pedigrees. Second, the effects of ethnic heterogeneity and sex difference on alcoholism have been extensively addressed. We selected non-Hispanic Caucasian males because they comprised the largest sample size among all subgroups. This single-ethnicity and sex-specific study sample, with a potential high risk, reduces genetic heterogeneity and potential false positive results; however, the small sample size may have hindered the detection of minor genetic effects and fit of complex genetic models, such as SNP–SNP and SNP–environment interactions. We attempted to include non-Hispanic Caucasian female founders and/or non-Hispanic black male founders in the pooled or replication study of the COGA data, but were obliged to give up since there were only 3 (4) alcoholism cases in 80 (8) Caucasian female (black male) founders having genotype data of the five selected SNPs from the mixture model. Hence, a family-based genetic association analysis is crucial and should be developed as a future work.

Population stratification should be evaluated and controlled carefully to avoid spurious genetic association. This study focuses on non-Hispanic Caucasian male founders in the COGA to reduce genetic heterogeneity. The study population is relatively homogeneous. We also performed principal component analysis to examine population stratification. Supplementary Figure S2(A) shows that the 65 non-Hispanic Caucasian males including 23 alcoholism cases and 42 unaffected subjects were mixed and separated from the non-Hispanic black male founders including 4 alcoholism cases and 4 unaffected subjects. Furthermore, Supplementary Figure S2(B) shows the non-Hispanic Caucasian males alcoholism cases and unaffected subjects were scattered randomly. Therefore, there is no need to adjust for population stratification in this study.

A final caution on using model selection and choosing modes of inheritance is taken to avoid leading to chance finding of the identification of the five SNPs for alcoholism. To validate the finite-sample reliability of our parameter estimation in the mixture model of single-SNP analysis, we employed bootstrap resampling method for regression models with censored data^45, 46^. The bootstrap validation results based on 400 bootstrap samples of size 65 are listed in Table 1(B). The bootstrap estimates are with the same magnitude of the parameter estimates in the original real data set, and 95% bootstrap percentile confidence intervals of the regression parameters, corresponding to the significant original estimates, do not contain zero; all indicating our fitted mixture models are reliable. We did not conduct bootstrap validation for multiple-SNPs analysis and gene-environment analysis since any resampling method applied to a sample of small size and many strata causes a high variation in the estimation. The bootstrap method provides a trustworthy way to estimate predictive accuracy of the mixture regression model via internal replications of non-Hispanic Caucasian male founders.

## Methods

### Data acquisition and ascertainment

The alcoholism data assembled for GAW14 were provided by the Collaborative Study on the Genetics of Alcoholism (U10 AA008401). Written informed consent was obtained from all subjects, and the institutional review boards of all six COGA collaborative sites approved all procedures. All experimental methods of the DNA collection, SNP genotyping and phenotypic characterization were performed in accordance with the relevant guidelines and regulations^19^. All subjects were diagnosed according to the COGA ascertainment criterion comprising the Diagnostic and Statistical Manual of Mental Disorders-III-R and Feighner criteria. An ascertainment procedure has been detailed^47^.

### Logistic-accelerated failure time location-scale mixture regression model

To facilitate the genotype analysis procedures, we used the web-based statistical software system EHA-RiskFree with a user friendly interface, which was developed according to the logistic-AFT location-scale mixture regression model^12^. We herein summarized the formulation, inference, and properties of the model.

Let *T* denote the age of onset of alcoholism; therefore, the survival distribution at *t* years of age Pr(*T* > *t*) indicates the probability that a subject is not affected with alcoholism by his age of *t* years. The study population comprised mixed subpopulations: affected and ultimately unaffected subjects. Let *D* denote the binary indicator of the development of alcoholism [*D* = 1 (yes) or 0 (no)]. The mixture regression model postulates that the probability that a subject with the risk factors *Z* and *Z* ^*^ has not developed alcoholism by his age of *t* years is as follows:

1 $$\Pr (T > t|Z,{Z}^{\ast })=\Pr (D=1|Z)\Pr (T > t|D=1,{Z}^{\ast })+\Pr (D=0|Z),$$

where *Z* and *Z* ^*^ denote the covariate vectors of SNPs or habitual smoking status for modelling the susceptibility and age of onset, respectively. Pr(*D* = 1|*Z*) is the probability of the eventual development of (or susceptibility to) alcoholism considering *Z*. The vectors *Z* and *Z* ^*^ with 1 as their first element may have some variables in common with other elements. In brief, the logistic-AFT mixture regression model simultaneously uses a logistic model to formulate the probability of alcoholism susceptibility and an AFT location-scale model to specify the conditional distribution Pr(*T* > *t*|*D* = 1, *Z* ^*^) of the alcoholism onset age for the susceptible subject. The logistic-AFT location-scale mixture model used in this study contains both a logistic regression submodel

2 $$\mathrm{ln}(\frac{\Pr (D=1|Z)}{\Pr (D=0|Z)})=\beta ^{\prime} Z$$

and an AFT location-scale regression submodel

3 $$\mathrm{ln}\,T({Z}^{\ast })=\gamma ^{\prime} {Z}^{\ast }+\exp (\alpha ^{\prime} {Z}^{\ast })\varepsilon ,$$

where $\mathrm{ln}\,T({Z}^{\ast })$ is the logarithmic transformation of *T* for the susceptible subject considering *Z* ^*^; *ε* is a specified class of error distributions corresponding to *Z* ^*^ = 0; and *β*, *γ*, and *α* are the corresponding parameters in the logistic, location, and scale regression parts, respectively. The entire mixture model defines the overall cumulative event probability as Pr(*T* ≤ *t*|*Z*, *Z* ^*^)  = 1 − Pr(*T* > *t*|*Z*, *Z* ^*^), and the AFT location-scale submodel defines the conditional cumulative event probability as Pr(*T* ≤ *t*|*D* = 1, *Z* ^*^) = 1 − Pr(*T* > *t*|*D* = 1, *Z* ^*^). The results of mixture regression modelling are reported as OR(*Z*) = exp(*β' Z*) with *γ* and *α* corresponding to each selected covariate in the tables.

The fitting results of the logistic, location, and scale regression parts in the logistic-AFT location-scale mixture model can be interpreted as follows. For each one-unit increase in *Z*, exp(*β*) is the odds ratio (OR) considering the probability Pr(*D* = 1|*Z*) of the eventual development of alcoholism for a subject with the factor *Z*. Hence, subjects with significant ORs of >1 and <1 tend to have a higher and lower probability to be susceptible to alcoholism development, respectively. Subjects with a positive *Z* ^*^ for significant *γ* > 0 and <0 tend to have a later and earlier age of onset, respectively. Moreover, subjects with a positive *Z* ^*^ for *α* > 0 tend to have a larger onset age range, whereas for *α* < 0 have a smaller onset age range.

We assumed that *T* of an alcoholism susceptible case follows a log-logistic distribution^48^ in the AFT location-scale regression submodel; in brief, error term *ε* has the logistic distribution of the density $f(\varepsilon )={e}^{\varepsilon }/{(1+{e}^{\varepsilon })}^{2}$. We fit a logistic regression submodel and an AFT log-logistic regression submodel to simultaneously study the associations of disease susceptibility and the age of onset with genetic and environmental effects.

### Associations between the identified SNPs and alcoholism

We first applied the mixture regression methodology to detect individual effects of the 22 SNPs on alcoholism in the 65 study subjects, according to the analysis flow chart (Supplementary Figure S3). Let the major and minor alleles be denoted as *A* and *a*, respectively, then *AA* is the major homozygous genotype, *Aa* is the heterozygous genotype, and *aa* is the minor homozygous genotype. In the model selection procedure for each SNP, because of the limited sample size, we first separated the SNPs into two subgroups: one is with an extremely rare alcoholism event (≤1) in any genotype and the other is with >1 alcoholism events in all genotypes.

SNPs of the extremely rare alcoholism event group corresponding to genotype *aa* include the following: (i) no-event subgroup in seven SNPs, namely rs157864, rs1157122, rs387661, rs1079597, rs1386493, rs1386492, and rs1838158 (with the last SNP having only two genotypes, *AA* and *Aa*), and (ii) one-event subgroup in three SNPs, namely rs1111418, rs927544, and rs2134655 (with only one subject having genotype *aa* of the last SNP and developing alcoholism. In these cases, we merged genotypes *aa* and *Aa* for appropriately handling sparse data in SNP analysis. In brief, we used a dummy variable to code *aa* = *Aa* = 1 and *AA* = 0 in the fitting of the mixture regression model.

The remaining 12 SNPs in this study were analysed using more options for model fitting. We performed simple regression on individual SNPs, first by using *Aa* as the reference genotype in the logistic, location, and scale regression parts. When the regression result shows two significant dummy variables of genotypes, it supports a general model with three genotypes (refer to the case of rs172677 in Fig. 1). Alternatively, for the remaining individual SNPs, we merged data of two genotypes whose event curves were closer among the three genotypes and subsequently estimated dominant or recessive effects by using the two subgroups based on the merged genotypes.

As shown in the flow chart (Supplementary Figure S3), we performed the likelihood ratio tests for fitted general, dominant, and recessive coding schemes compared with the null model in the mixture regression with different degrees of freedom. We used the minimum Akaike information criterion to select the best model from a subset of candidate models, including logistic regression submodels (with only a constant intercept in the AFT submodel), AFT location-scale regression submodels (with only a constant intercept in the logistic submodel), and logistic-AFT location-scale mixture regression models.

To visualize the adequacy of the best-fitted model for each SNP, we compared the smooth overall event curves and the smooth conditional event curves of susceptible cases estimated from the mixture regression model with the step-function overall and conditional event curves computed using the Kaplan–Meier estimator^20^ among the studied genotypes. In addition to these intuitive plots, the residual plots for the mixture regression model^12^ were investigated but not included in this report because of their satisfactory performances and sophistications. The plots indicate that the assumed log-logistic distribution well fits the error term in the AFT model for the COGA data set.

### GAP clustering of the identified SNP–environment associations and pathway analysis

GAP is an exploratory data analysis software for matrix visualization and clustering of high-dimensional data sets^25, 26^. To achieve a direct visual perception of the relationship among selected SNPs and environmental covariates, we first defined a binary data matrix, **B**, to describe the relationship between subjects and genes/smoking. In **B**, each row indicates a subject, and each column indicates a SNP or the habitual smoking status. The element on the *r-*th row and *s-*th column, **B**_*rs*_, was coded as 1 with black if the *r-*th subject had genotype *Aa* or *aa* of the *s-*th SNP or was a habitual smoker; otherwise, the element was coded as 0 with white. The Jaccard coefficient (0, no similarity and 1, complete similarity) was used to measure both between-subject associations and between-gene/smoking associations as in **G** and **P**. These three matrices, **G** (subjects), **P** (genes/smoking), and **B** (subjects by genes/smoking), were subsequently sorted by the average-linkage hierarchical clustering tree with the flipping of intermediated nodes guided through elliptical seriation^49^ for identifying subject clusters and gene/smoking-subgroups with interactions between them. The alcoholism onset status and corresponding onset and follow-up age are shown as covariates to the left of **B**.

We also analysed pathways containing the studied alcoholism genes from KEGG^27^. Similar to the exploration of the relationship between subjects and genes/smoking, subjects and genes/smoking interaction, GAP was used to study the gene–pathway interactions on the basis of a binary data matrix **B**, with **G** (genes) and **P** (pathways) measured using the Jaccard coefficient. In **B**, each row indicates SNPs in a gene, and each column indicates a pathway named using hierarchical categories, separated by semicolons in order, as defined in the KEGG. The five categories in the KEGG pathway maps are metabolism, environmental information processing, cellular processes, organismal systems, and human diseases. Moreover, **G**, **P**, and **B**, were sorted by the same GAP procedure as aforementioned for identifying gene-clusters and pathway-groups with interactions between them. The pathway categories are displayed as a covariate on the top of **B** and to the left of **P**.

## Electronic supplementary material

Supplementary Information

## References

[1] Altshuler D, Daly MJ, Lander ES (2008). Genetic mapping in human disease. Science.

[2] Collins A, Morton NE (1998). Mapping a disease locus by allelic association. Proc. Natl. Acad. Sci. USA.

[3] Elston RC (1998). Linkage and association. Genet. Epidemiol..

[4] Burton PR (2007). Genome-wide association study of 14,000 cases of seven common diseases and 3,000 shared controls. Nature.

[5] Cox, D. R. & Snell, E. J. *Analysis of Binary Data*, 2nd edn. (Chapman and Hall, 1989).

[6] Sasieni PD (1997). From genotypes to genes: Doubling the sample size. Biometrics.

[7] Chiano MN, Clayton DG (1998). Fine genetic mapping using haplotype analysis and the missing data problem. Ann. Hum. Genet..

[8] Cordell HJ, Clayton DG (2002). A unified stepwise regression procedure for evaluating the relative effects of polymorphisms within a gene using case/control or family data: Application to HLA in type 1 diabetes. Am. J. Hum. Genet..

[9] Aalen, O., Borgan, O. & Gjessing, H. Survival and Event History Analysis: A Process Point of View (Springer, 2008).

[10] Keiding N (2014). Event history analysis. Annu. Rev. Stat. Appl..

[11] Cox DR (1972). Regression models and life tables (with discussion). J. R. Stat. Soc. Ser. B.

[12] Chen CH, Tsay YC, Wu YC, Horng CF (2013). Logistic-AFT location-scale mixture regression models with non-susceptibility for left-truncated and general interval-censored data. Stat. Med..

[13] Stringer S, Denys D, Kahn RS, Derks EM (2016). What cure models can teach us about genome-wide survival analysis. Behav. Genet..

[14] Mayfield RD, Harris RA, Schuckit MA (2008). Genetic factors influencing alcohol dependence. Br. J. Pharmacol..

[15] Hasin DS, Stinson FS, Ogburn E, Grant BF (2007). Prevalence, correlates, disability, and comorbidity of DSM-IV alcohol abuse and dependence in the United States: results from the National Epidemiologic Survey on Alcohol and Related Conditions. Arch. Gen. Psychiatry.

[16] Prescott CA, Kendler KS (1999). Genetic and environmental contributions to alcohol abuse and dependence in a population-based sample of male twins. Am. J. Psychiatry.

[17] Blum K (1996). (1996) The D2 dopamine receptor gene as a determinant of reward deficiency syndrome. J. R. Soc. Med..

[18] Luo X (2005). CHRM2 gene predisposes to alcohol dependence, drug dependence and affective disorders: results from an extended case-control structured association study. Hum. Mol. Genet..

[19] Edenberg HJ (2005). Description of the data from the Collaborative Study on the Genetics of Alcoholism (COGA) and single-nucleotide polymorphism genotyping for Genetic Analysis Workshop 14. BMC Genet..

[20] Kaplan EL, Meier P (1958). Nonparametric estimation from incomplete observations. J. Am. Stat. Assoc..

[21] McKusick-Nathans Institute of Genetic Medicine, Johns Hopkins University. Online Mendelian Inheritance in Man. http://omim.org/ (Date of access: 07/02/2014).

[22] Becker, K. G., Barnes, K. C., Bright T. J. & Wang, S. A. The Genetic Association Database. *Nat. Genet*. **36**, 431–432 http://geneticassociationdb.nih.gov/ (Date of access: 07/02/2014) (2004). 10.1038/ng0504-431 15118671

[23] Smigielski, E. M., Sirotkin, K., Ward, M. & Sherry, S. T. dbSNP: a database of single nucleotide polymorphisms. *Nucleic Acids Res*. **28**, 352–355 http://www.ncbi.nlm.nih.gov/SNP/ (Date of access:07/02/2014) (2000). 10.1093/nar/28.1.352 PMC102496 10592272

[24] Barrett JC, Fry B, Maller J, Daly MJ (2005). Haploview: analysis and visualization of LD and haplotype maps. Bioinformatics.

[25] Chen CH (2002). Generalized Association Plots: Information visualization via iteratively generated correlation matrices. Stat. Sinica..

[26] Wu HM, Tien YJ, Chen CH (2010). GAP: A graphical environment for matrix visualization and cluster analysis. Comput. Stat. Data An..

[27] Kanehisa, M. & Goto, S. KEGG: Kyoto Encyclopedia of Genes and Genomes. *Nucleic Acids Res*. **28**, 27–30 http://www.genome.jp/kegg/ (Date of access: 07/02/2014) (2000). 10.1093/nar/28.1.27 PMC102409 10592173

[28] Hallikainen T (1999). Association between low activity serotonin transporter promoter genotype and early onset alcoholism with habitual impulsive violent behavior. Mol. Psychiatry.

[29] Wiener HW, Go RC, Tiwari H, George V, Page GP (2005). COGA phenotypes and linkages on chromosome 2. BMC Genet..

[30] Feng R, Zhang H (2007). A score test for linkage analysis of ordinal traits based on IBD sharing. Biostatistics.

[31] Liu IC (2004). Genetic and environmental contributions to age of onset of alcohol dependence symptoms in male twins. Addiction.

[32] Li YJ, Martin ER, Zhang L, Allen AS (2005). Application of a rank-based genetic association test to age-at-onset data from the Collaborative Study on the Genetics of Alcoholism study. BMC Genet..

[33] Tayo BO, Liang Y, Stranges S, Trevisan M (2005). Genome-wide linkage analysis of age at onset of alcohol dependence: a comparison between microsatellites and single-nucleotide polymorphisms. BMC Genet..

[34] Zhong X, Zhang H (2005). Linkage analysis and association analysis in the presence of linkage using age at onset of COGA alcoholism data. BMC Genet..

[35] Zhao JH (2005). Mixed-effects Cox models of alcohol dependence in extended families. BMC Genet..

[36] Kapoor M (2014). Genome-wide survival analysis of age at onset of alcohol dependence in extended high-risk COGA families. Drug Alcohol Depend..

[37] Blum K (1993). Genetic predisposition in alcoholism: Association of the D2 dopamine receptor TaqI B1 RFLP with severe alcoholics. Alcohol.

[38] Traynelis, S. F. *et al*. Glutamate receptor ion channels: structure, regulation, and function. *Pharmacol. Rev*. **62**, 405–496 (2010). Erratum **66**, 1141 (2014). 10.1124/pr.109.002451 PMC2964903 20716669

[39] Gécz J (2010). Glutamate receptors and learning and memory. Nat. Genet..

[40] Nagy J (2008). Alcohol related changes in regulation of NMDA receptor functions. Curr. Neuropharmacol..

[41] Yoshida A, Davé V, Ward RJ, Peters TJ (1989). Cytosolic aldehyde dehydrogenase (ALDH1) variants found in alcohol flushers. Ann. Hum. Genet..

[42] Konishi T (2004). *ADH1B*1, ADH1C*2, DRD2* (-141C Ins), and *5-HTTLPR* are associated with alcoholism in Mexican American men living in Los Angeles. Alcohol Clin. Exp. Res..

[43] Lange K (2013). Mendel: The Swiss army knife of genetic analysis programs. Bioinformatics.

[44] S.A.G.E. http://darwin.cwru.edu (2016).

[45] Efron B (1981). Censored data and the bootstrap. J. Am. Stat. Assoc..

[46] Chen C-H, George SL (1985). The bootstrap and identification of prognostic factors via Cox’s proportional hazards regression model. Stat. Med..

[47] Begleiter H (1999). Description of the Genetic Analysis Workshop 11 Collaborative Study on the Genetics of Alcoholism. Genet. Epidemiol..

[48] Bennett S (1983). Log-logistic regression models for survival data. J. R. Stat. Soc. Ser. C (Appl. Stat.).

[49] Tien YJ, Lee YS, Wu HM, Chen CH (2008). Methods for simultaneously identifying coherent local clusters with smooth global patterns in gene expression profiles. BMC Bioinformatics.

[50] Lewontin RC (1995). The detection of linkage disequilibrium in molecular sequence data. Genetics.

[51] Gabriel SB (2002). The structure of haplotypes in the human genome. Science.

