# Supplementary material for: Using an Event-History with Risk-Free Model to Study the Genetics of Alcoholism
Source: Sci Rep. 2017 May 16;7:1975. doi: 10.1038/s41598-017-01791-4 (PMC5434012; doi:10.1038/s41598-017-01791-4)

Supplementary Information

Using an Event-History with Risk-Free Model to Study the Genetics of Alcoholism

Hsin-Chou Yang[1], I-Chen Chen[1,2], Yuh-Chyuan Tsay[1], Zheng-Rong Li[1], Chun-houh Chen[1], Hai-Gwo Hwu[3,4] & Chen-Hsin Chen[1,3,*]

[1]Institute of Statistical Science, Academia Sinica, Taipei 11529, Taiwan. [2]Department of Biostatistics, University of Kentucky, Lexington, KY 40506, U.S.A. [3]Graduate Institute of Epidemiology and Preventive Medicine, National Taiwan University, Taipei, Taiwan. [4]Department of Psychiatry, National Taiwan University Hospital and College of Medicine, National Taiwan University, Taipei, Taiwan. [*]Correspondence and requests for materials should be addressed to C.-H.C. (e-mail: chchen@stat.sinica.edu.tw)

Supplementary List S1: Fifteen genes collected from OMIM

The following 15 genes associated with alcoholism were collected from OMIM: *ADH1B*, *ADH1C*, *ALDH2*, *GABRA2*, *GABRA4*, *GABRA6*, *GABRB1*, *GABRG1*, *NPY*, *TAS2R16*, *CHRM2*, *DRD2*, *ANKK1*, *SLC6A4*, and *COMT*.

Supplementary List S2: Sixty-five genes collected from GAD

The 65 genes associated with alcoholism collected from GAD are as follows: *ACE, ADAM11*, *ADD1, ADH1B, ADH1C, ADRA2C, AGT*, *ALDH1A1*, *ALDH2, ARSA*, *CART*, *CCK*, *CCKAR*, *CD14*, *CEL*, *CHRNA4*, *CNR1*, *COL1A1*, *COMT*, *CRHR1*, *CYP2E1*, *DBH*, *DRD1*, *DRD2*, *DRD3*, *DRD4*, *FYN*, *GABRA1*, *GABRA2*, *GABRA6*, *GABRB1*, *GABRB2*, *GABRB3*, *GABRG2*, *GAD2*, *GAL*, *GALR1*, *GALR2*, *GALR3*, *GRIN2B*, *HNMT*, *HTR1B*, *HTR2A*, *HTR2C*, *IL1RN*, *LPRP*, *MAOA*, *MGLL*, *NPY*, *NR4A2*, *NTRK2*, *OPRM1*, *P450IIE1*, *PAP*, *RARA*, *RARB*, *RARG*, *RXRA*, *RXRB*, *RXRG*, *SLC6A3*, *SLC6A4*, *TH*, *TPH1*, and *TPH2*.

Supplementary Table S1. The nine excluded SNPs. Abbreviations: GCR, genotyping call rate; MAF, minor allele frequency; HWE, Hardy-Weinberg equilibrium test.

Chromosome	Gene	RS ID	Position	Chr. Position	Category	GCR	MAF	HWE p-value
4	*GABRB1*	rs728293	65.9838	47357683	intron	100	0.485	0.029
4	*GABRB1*	rs956412	65.9839	47357850	intron	100	0.485	0.032
6	*FYN*	rs1409836	116.07	112169350	intron	95.4	0	0.779
6	*FYN*	rs910683	116.07	112183287	intron	100	0.008	1
11	*DRD2*	rs1079598	112.859	113296274	intron	98.5	0.133	1
11	*DRD2*	rs1079596	112.859	113296619	intron	100	0.131	1
12	*GRIN2B*	rs1421109	31.4933	14132811	intron	73.8	0.031	1
12	*TPH2*	rs1386483	86.4294	72412494	intron	96.9	0.341	0.09
13	*HTR2A*	rs985934	50.6279	47455725	intron	98.5	0.367	0.526

Supplementary Table S2. The 22 studied SNPs on 12 final candidate genes. Abbreviations: GCR, genotyping call rate; MAF, minor allele frequency; HWE, Hardy-Weinberg equilibrium test.

Chromosome	Gene	RS ID	Position	Chr. Position	Category	GCR	MAF	HWE p-value
1	*RXRG*	rs2134095	165.75	165377552	cds-synon	100	0.338	1
1	*RXRG*	rs157864	165.757	165383761	intron	96.9	0.143	1
3	*DRD3*	rs2134655	121.807	113858201	intron	100	0.2	0.583
5	*GABRA1*	rs966137	165.501	161315096	intron	84.6	0.264	1
5	*GABRA1*	rs1157122	165.505	161319314	intron	100	0.092	0.376
5	*GABRG2*	rs411409	165.669	161543571	intron	86.2	0.384	1
5	*GABRG2*	rs387661	165.669	161543711	intron	96.9	0.167	0.236
5	*GABRG2*	rs2422106	165.682	161561803	intron	100	0.338	1
7	*CHRM2*	rs1378647	141.86	136633183	intron	100	0.323	1
7	*CHRM2*	rs1111418	141.859	136633704	intron	100	0.292	0.072
9	*ALDH1A1*	rs63319	68.3967	75524784	intron	87.7	0.447	0.532
9	*ALDH1A1*	rs348457	68.3986	75530554	intron	100	0.392	0.532
9	*NTRK2*	rs1439047	84.06	87327918	intron	83.1	0.435	0.494
9	*NTRK2*	rs1838158	84.06	87473009	intron	98.5	0.133	0.573
11	*DRD2*	rs1079597	112.859	113296286	intron	100	0.131	1
12	*GRIN2B*	rs172677	31.454	14097470	intron	100	0.408	0.343
12	*TPH2*	rs1386493	86.4162	72355179	intron	100	0.138	0.523
12	*TPH2*	rs1386492	86.4179	72362265	intron	93.8	0.148	0.124
12	*TPH2*	rs1386485	86.4294	72412367	intron	98.5	0.352	0.183
13	*HTR2A*	rs985933	50.6281	47455863	intron	100	0.369	0.518
13	*HTR2A*	rs927544	50.6283	47456051	intron	96.9	0.294	0.495
15	*GABRB3*	rs1365225	11.3281	26944619	intron	100	0.323	0.729

Supplementary Table S3. Analysis of the logistic-AFT mixture regression model stratified by the habitual smoking status with chi-squared statistic 4.50 (p = 0.034) of the likelihood ratio test in the mixture model. Abbreviations: OR, odds ratio; CI, confidence interval; HS, Habitual smoking (Yes=1, No=0).

Covariate	Logistic Regression Submodel			AFT Submodel (Log-logistic Event Time Distribution)					
				Location Regression Part			Scale Regression Part		
	OR	95% CI	p-value	Estimate	95% CI	p-value	Estimate	95% CI	p-value
Intercept	1		Referent	3.77	3.32, 4.22	Referent	-1.57	-1.97, -1.17	Referent
HS	1			-0.47	-0.94, -0.01	0.046	0		

Supplementary Table S4. Single SNP analysis using the conventional logistic regression model and the conventional Cox PH regression model. [a] The sample size for the corresponding single-gene analysis. [b] Chi-squared statistic and p-value for the likelihood ratio test. Abbreviations: OR, odds ratio; CI, confidence interval; HR, hazard ratio; LRT, likelihood ratio test.

SNP [Gene]	Covariates	Conventional Logistic Regression			LRT[b]		Conventional Cox PH Regression			LRT[b]	
(# Subjects)[a]	(Genotypes)	OR	95% CI	p-value	χ^2	p-value	HR	95% CI	p-value	χ^2	p-value
rs172677	Intercept	1		Referent (*Aa*)	0.02	0.991	1		Referent (*Aa*)	0.16	0.923
[*GRIN2B*] (65)	*aa*	1.11	0.23, 5.39	0.899			1.22	0.35, 4.28	0.757		
	AA	0.99	0.32, 3.11	0.992			0.92	0.37, 2.31	0.858		
rs172677	Intercept	1		Referent (*AA*)	0.01	0.919	1		Referent (*AA*)	0.14	0.705
[*GRIN2B*] (65)	*a* (*Aa*=1, *aa*=2)	1.04	0.47, 2.34	0.919			1.14	0.59, 2.18	0.704		
rs1439047	Intercept	1		Referent (*AA*)	0.03	0.859	1		Referent (*AA*)	0.05	0.825
[*NTRK2*] (54)	*Aa* + *aa*	1.12	0.32, 3.94	0.860			0.89	0.32, 2.48	0.823		
rs2134655	Intercept	1		Referent (*AA*)	4.87	0.027	1		Referent (*AA*)	4.54	0.033
[*DRD3*] (65)	*Aa* + *aa*	3.25	1.12, 9.40	0.030			2.45	1.07, 5.60	0.034		
rs63319	Intercept	1		Referent (*AA*)	0.97	0.324	1		Referent (*AA*)	0.58	0.448
[*ALDH1A1*] (57)	*Aa* + *aa*	0.56	0.18, 1.77	0.323			0.70	0.28, 1.74	0.442		
rs1079597	Intercept	1		Referent (*AA*)	2.76	0.120	1		Referent (*AA*)	3.02	0.082
[*DRD2*] (65)	*Aa* + *aa*	0.34	0.08, 1.33	0.120			0.38	0.11, 1.29	0.121		

Supplementary Figure S1. Kaplan–Meier (step function) and the mixture regression (smooth curve) estimators of overall and conditional event curves for the alcoholism onset age stratified by habitual smoking status

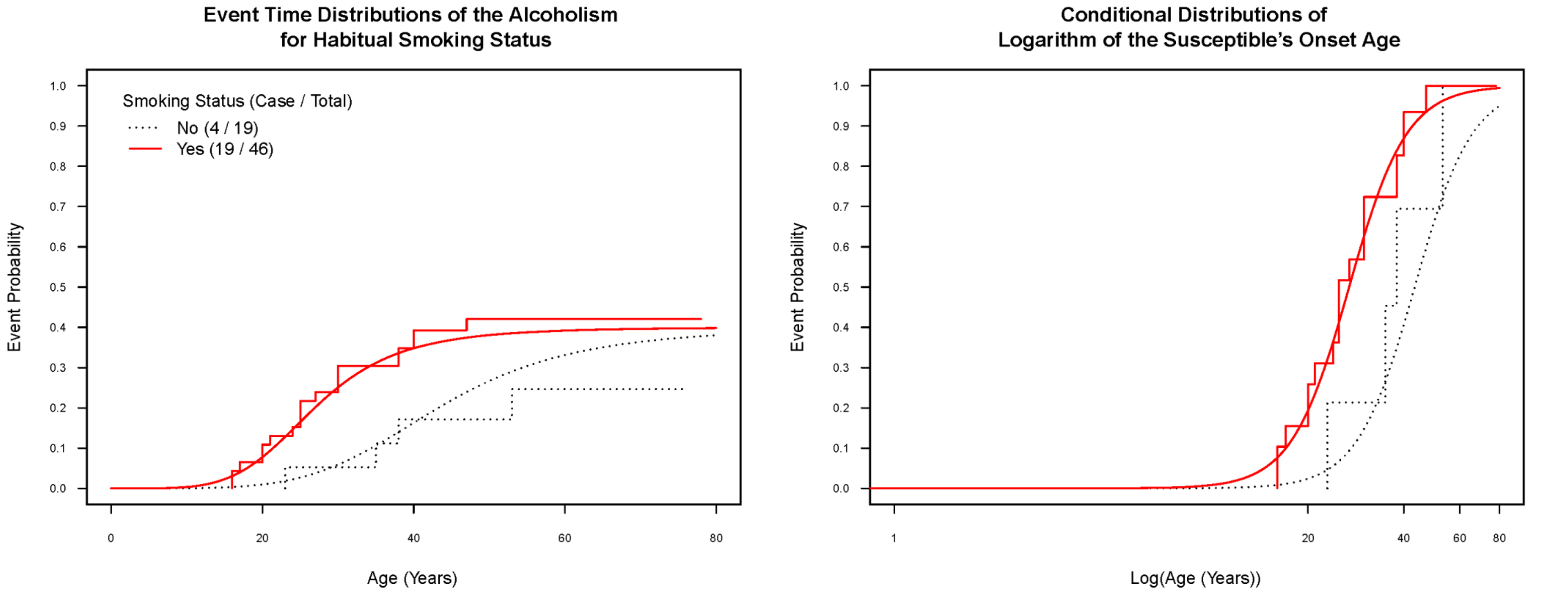

Supplementary Figure S2. Population stratification analysis. (A) Principal component (PC) plot of the non-Hispanic Caucasian male founders and non-Hispanic black male founders based on the whole-genome SNPs. (B) PC plot of the non-Hispanic Caucasian male founders based on the whole-genome SNPs. The dashed ellipse indicates the 99% confidence band of the first two principal components.

(A)

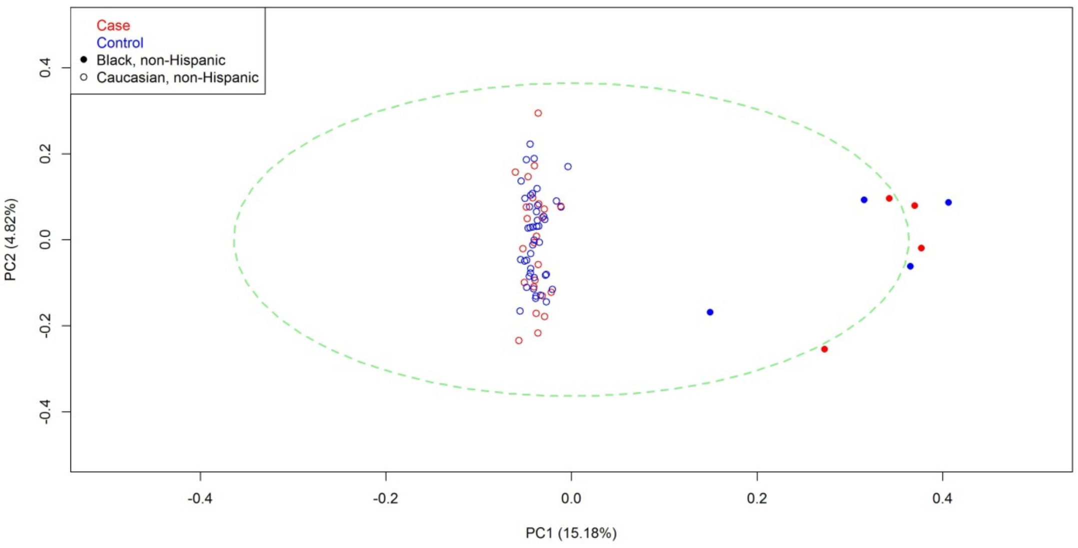

(B)

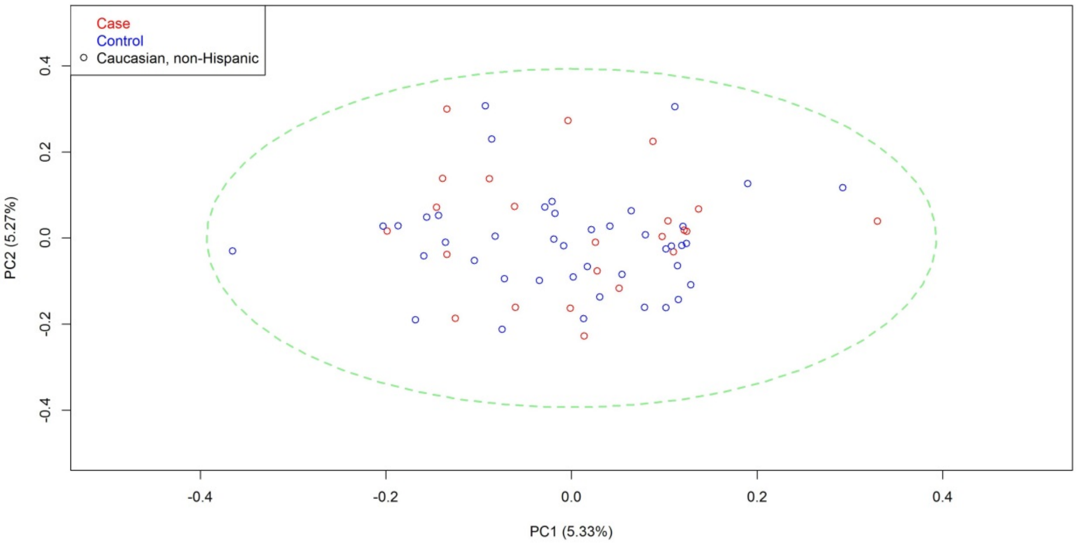

Supplementary Figure S3. Flow chart and the corresponding SNPs in categories of the genotype analyses

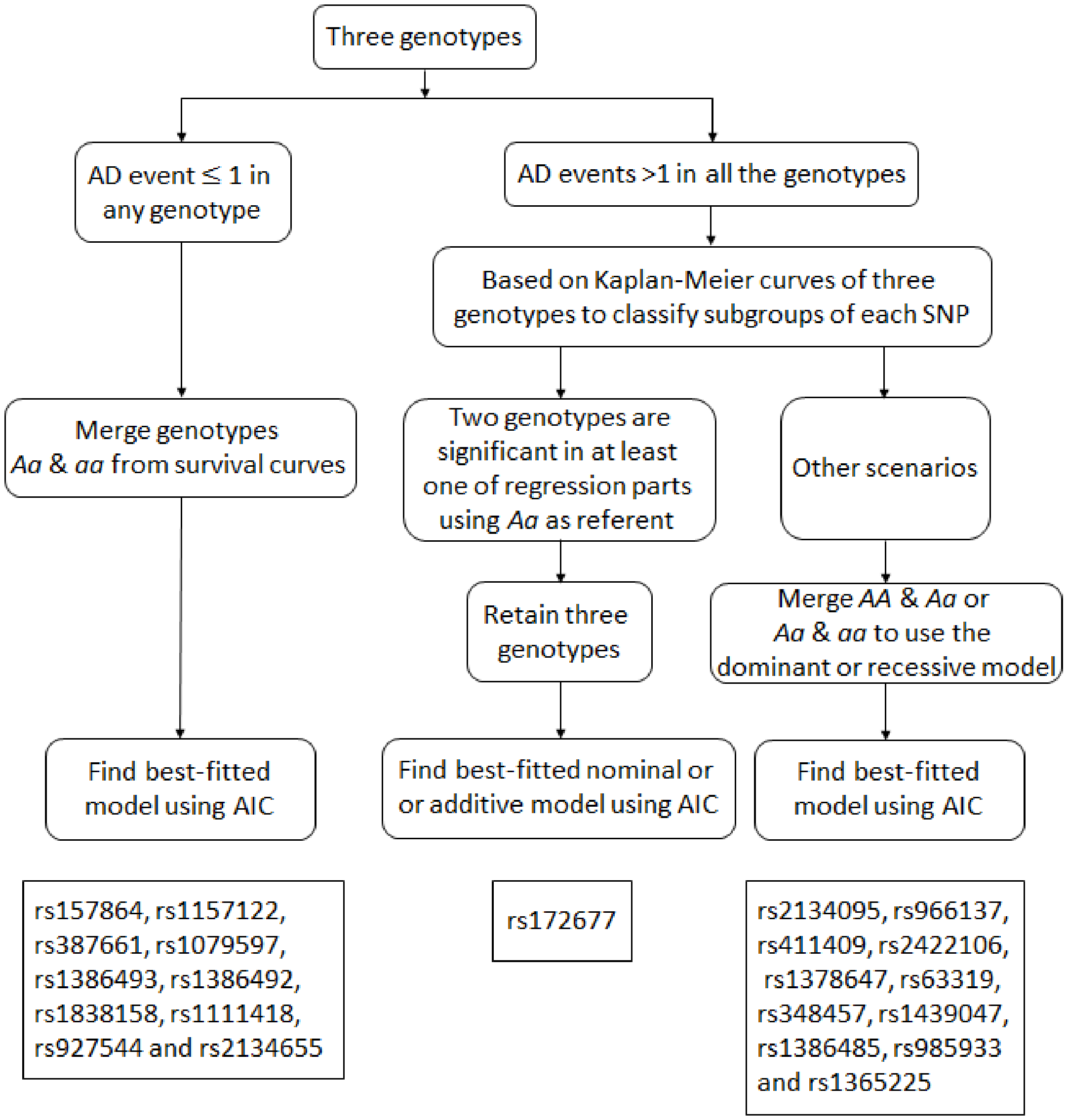

Supplement: Supplementary file 1 — Supplementary Information [file 41598_2017_1791_MOESM1_ESM.pdf]
